# Meclozine Facilitates Proliferation and Differentiation of Chondrocytes by Attenuating Abnormally Activated FGFR3 Signaling in Achondroplasia

**DOI:** 10.1371/journal.pone.0081569

**Published:** 2013-12-04

**Authors:** Masaki Matsushita, Hiroshi Kitoh, Bisei Ohkawara, Kenichi Mishima, Hiroshi Kaneko, Mikako Ito, Akio Masuda, Naoki Ishiguro, Kinji Ohno

**Affiliations:** 1 Division of Neurogenetics, Center for Neurological Diseases and Cancer, Nagoya University Graduate School of Medicine, Nagoya, Japan; 2 Department of Orthopaedic Surgery, Nagoya University Graduate School of Medicine, Nagoya, Japan; SERGAS, Santiago University Clinical Hospital, IDIS Research Laboratory 9, NEIRID Lab, Spain

## Abstract

Achondroplasia (ACH) is one of the most common skeletal dysplasias with short stature caused by gain-of-function mutations in FGFR3 encoding the fibroblast growth factor receptor 3. We used the drug repositioning strategy to identify an FDA-approved drug that suppresses abnormally activated FGFR3 signaling in ACH. We found that meclozine, an anti-histamine drug that has long been used for motion sickness, facilitates chondrocyte proliferation and mitigates loss of extracellular matrix in FGF2-treated rat chondrosarcoma (RCS) cells. Meclozine also ameliorated abnormally suppressed proliferation of human chondrosarcoma (HCS-2/8) cells that were infected with lentivirus expressing constitutively active mutants of FGFR3-K650E causing thanatophoric dysplasia, FGFR3-K650M causing SADDAN, and FGFR3-G380R causing ACH. Similarly, meclozine alleviated abnormally suppressed differentiation of ATDC5 chondrogenic cells expressing FGFR3-K650E and -G380R in micromass culture. We also confirmed that meclozine alleviates FGF2-mediated longitudinal growth inhibition of embryonic tibia in bone explant culture. Interestingly, meclozine enhanced growth of embryonic tibia in explant culture even in the absence of FGF2 treatment. Analyses of intracellular FGFR3 signaling disclosed that meclozine downregulates phosphorylation of ERK but not of MEK in FGF2-treated RCS cells. Similarly, meclozine enhanced proliferation of RCS cells expressing constitutively active mutants of MEK and RAF but not of ERK, which suggests that meclozine downregulates the FGFR3 signaling by possibly attenuating ERK phosphorylation. We used the C-natriuretic peptide (CNP) as a potent inhibitor of the FGFR3 signaling throughout our experiments, and found that meclozine was as efficient as CNP in attenuating the abnormal FGFR3 signaling. We propose that meclozine is a potential therapeutic agent for treating ACH and other FGFR3-related skeletal dysplasias.

## Introduction

Achondroplasia (ACH) is one of the most common skeletal dysplasias with an incidence of one in 16,000 to 26,000 live births [1]. Clinical features of ACH include rhizomelic short stature, apparent macrocephaly with midface hypoplasia, bowing of the lower limbs, and increased lumbar lordosis [2]. ACH is caused by gain-of-function mutations in the fibroblast growth factor receptor 3 (*FGFR3*) gene [3], [4]. FGFR3 is a key regulator of endochondral bone growth, which signals through several intracellular pathways including the signal transducer and activator of transcription (STAT) and mitogen-activated protein kinase (MAPK) [5]–[7]. Gain-of-function mutations of *FGFR3* cause several short-limbed skeletal dysplasias such as hypochondroplasia (HCH) [8], severe ACH with developmental delay and acanthosis nigricans (SADDAN) [9], and thanatophoric dysplasia (TD) types I and II [10]. In contrast, loss of function mutations in *FGFR3* lead to the CATSHL syndrome in humans, which is characterized by overgrowth of the skeleton including camptodactyly, tall stature, scoliosis, and hearing loss [11], as well as spider lamb syndrome in sheep [12]. These findings indicate that the FGFR3 signaling functions as a negative regulator of endochondral bone growth.

No effective treatments for FGFR3-related skeletal dysplasias are currently available. Growth hormone (GH) has been administered to children with ACH based on evidence of a short-term beneficial effect [13]. The response to GH, however, is moderate and the long-term effect remains controversial. It is conceivable that downregulation of the FGFR3 signaling alleviates the skeletal phenotype of FGFR3-related skeletal dysplasias. Small chemical compounds that antagonize the FGFR3 signaling have recently been identified. Toxicological profiles of these compounds, however, remain mostly unresolved [14]-[16]. The C-type natriuretic peptide (CNP) is a potent antagonist of the FGFR3 signaling that alleviates the short-limbed phenotype of ACH mice through its inhibition of the FGFR3-MAPK pathway [6], [17]. CNP has a short half-life and continuous intravenous infusion is required for *in vivo* experiments [18]. The CNP analog with an extended half-life, BMN 111, has recently been developed and significant recovery of bone growth was demonstrated in ACH mice by subcutaneous administration of BMN 111 [19].

The drug repositioning strategy, in which a drug currently used for patients with a specific disease is applied to another disease, has gained increasing attention from both academia and industry in recent years [20], [21]. The advantage of this strategy is that the identified drugs can be readily applied to clinical practice, because the optimal doses and adverse effects are already established. Here, we screened 1,186 FDA-approved compounds to identify a clinically applicable drug that ameliorates ACH and other FGFR3-related skeletal dysplasias. We found that meclozine dihydrochloride, a commonly used anti-emetic drug for its anti-histamine activity, efficiently suppresses FGFR3 signaling in three different chondrocytic cell lines and embryonic bone organ culture. We also identified that meclozine suppresses FGF2-mediated phosphorylation of ERK.

## Results

### Meclozine facilitates chondrocyte proliferation and mitigates loss of extracellular matrix in FGF2-treated RCS cells

**Figure 1:**
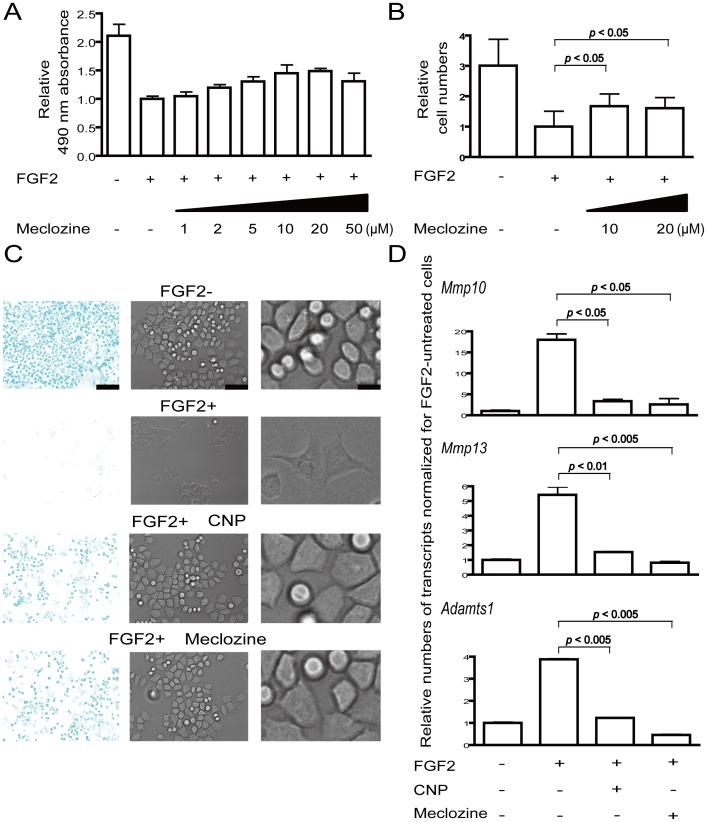
Meclozine promotes chondrocyte proliferation and ameliorates loss of extracellular matrix in FGF2-treated RCS cells. (A, B) RCS cells were treated with 5 ng/ml FGF2 and the indicated concentrations of meclozine for 48 hours. Cell growth was quantified using the MTS assay (A) or by counting cells (B). Data are normalized to that without meclozine and indicated by the mean and SD (*n* = 8 for A and 6 for B). Meclozine rescued the FGF2-mediated growth arrest of RCS cells. (C) Meclozine (10 µM) ameliorated FGF2-mediated alteration of cellular shape and loss of extracellular matrix. RCS cells were treated with 5 ng/ml FGF2 with and without 0.2 µM CNP or 20 µM meclozine for 72 hours, and cartilage-like sulfated proteoglycan matrix was stained by Alcian blue. Growing RCS cells were round-shaped and produced abundant cartilage-like sulfated proteoglycan matrix in the absence of FGF2. FGF2 treatment transformed some cells to fibroblast-like shapes and prominently suppressed expression of sulfated proteoglycan matrix. In the RCS cells treated with CNP or meclozine, the cellular shape remained round and the intensity of Alcian blue staining approximated that of FGF2-negative cells. Representative images of triplicated experiments are shown. Magnified images of the middle panels are shown in the rightmost column. Bars in the left, middle, and right panels are 750, 150, and 30 µm, respectively. (D) Meclozine (20 µM) inhibited mRNA expression of matrix metalloproteinases in FGF2-treated RCS cells. Cells were treated with FGF2 and either CNP or meclozine for four hours and mRNAs were quantified by real-time RT-PCR. Expression levels of *Mmp10*, *Mmp13*, and *Adamts1* are presented as the mean and SD normalized to that of FGF2-negative cells (*n* = 3). FGF2-mediated increases of *Mmp10*, *Mmp13*, and *Adamts1* mRNA were antagonized by CNP and meclozine. Statistical significance is estimated by Student's t-test.

As rat chondrosarcoma (RCS) chondrocytic cells express high levels of FGFR3, exogenous administration of FGF2 readily recapitulates cellular processes occurring in FGFR3-related skeletal dysplasias [22]. We thus added 10 µM of 1,186 FDA-approved chemical compounds (Prestwick Chemical) along with 5 ng/ml FGF2 to the RCS cells. Quantification of RCS proliferation by the MTS assay revealed that meclozine consistently induced 1.4-fold or more increases in RCS proliferation. In addition, 0, 1, 2, 5, 10, and 20 µM of meclozine exhibited dose-dependent increases in RCS proliferation (Figure 1A). We did not observe dose-dependency at 50 µM, which was likely due to cell toxicity. We also confirmed that 10 and 20 µM of meclozine increased the number of RCS cells (Figure 1B).

We next compared the effect of meclozine with that of CNP [6], [17] as a positive control. Growing RCS cells produced a large amount of cartilage-like sulfated proteoglycans, which were visualized by Alcian blue staining (Figure 1C). The matrix proteoglycans were almost completely lost at 72 hours after addition of FGF2 due to inhibition of proteoglycan production and also to induction of matrix metalloproteinase-mediated degradation [6]. Treatment of FGF2-added RCS cells with meclozine partly restored staining for Alcian blue and round chondrocyte-like cell shapes, which were similar to those observed with CNP.

We first confirmed that treating RCS cells with FGF2 for four hours induced expressions of matrix metalloproteinase 10 (*Mmp10*), *Mmp13*, and a disintegrin-like and metalloproteinase with thrombospondin type 1 motif 1 (*Adamts1*) transcripts, as has been previously reported [6]. We found that meclozine and CNP significantly suppressed expressions of these matrix metalloproteinases (Figure 1D). We also quantified expressions of *Col2a1* and *Acan* transcripts, but FGF2 treatment for 72 hours did not reduce the expression levels of these genes in RCS cells (Figure S1). Meclozine thus decreases expressions of the matrix metalloproteinases in FGF2-treated RCS cells.

### Meclozine mitigates abnormally suppressed proliferation of HCS-2/8 chondrocytes expressing FGFR3-K650E, -K650M, and -G380R

**Figure 2:**
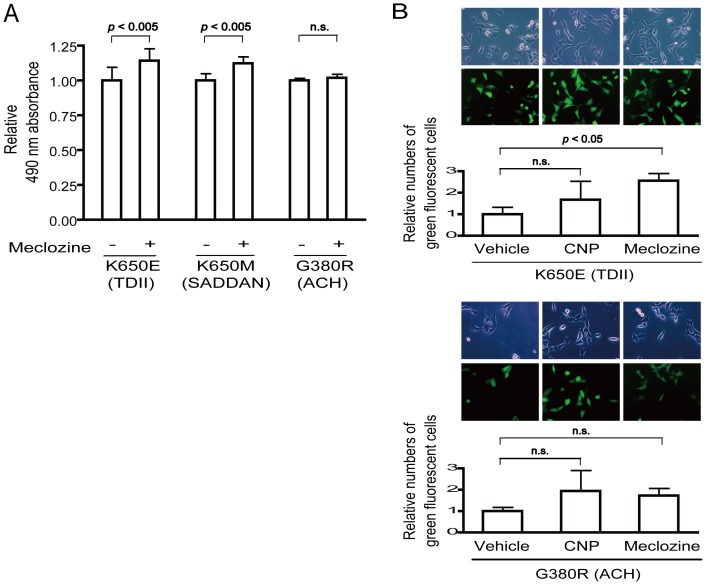
Meclozine mitigates abnormally suppressed proliferation of HCS-2/8 chondrocytes infected with lentivirus expressing mutant FGFR3. (A) HCS-2/8 cells expressing either FGFR3-K650E causing TDII, -K650M causing SADDAN, or G380R causing ACH were cultured with 20 µM meclozine for 48 hours and the proliferation was quantified by the MTS assay. Values are normalized to that without meclozine and the mean and SD are presented (*n* = 12). Meclozine ameliorated growth arrest driven by the mutations. (B) CNP (0.2 µM) and meclozine (20 µM) also alleviated K650E- and G380R-mediated growth inhibition visualized by Venus fluorescence expressed by the infected lentivirus. Fluorescence microscopy shows that the transfection efficiency is more than 90%. Both CNP and meclozine increased the Venus signal areas. Data are presented as the mean and SE that are normalized to that of vehicle (*n* = 3). Statistical significance is estimated by Student's t-test.

We examined the effects of meclozine on chondrocyte proliferation under the influence of FGFR3 mutants. As RCS cells express high levels of wild-type FGFR3, we used human chondrosarcoma (HCS-2/8) cells to observe unequivocal effects of the transduced FGFR3 mutants. We first introduced lentivirus carrying active mutants of FGFR3 (K650E in TDII, K650M in SADDAN, and G380R in ACH) into HCS-2/8 cells. The lentivirus carried IRES2-driven Venus cDNA downstream of FGFR3 cDNA. The MTS assay demonstrated that K650E-expressing HCS-2/8 cells showed significantly suppressed cellular proliferation (Figure S2). Meclozine partially rescued the growth arrest without apparent cellular toxicity in HCS-2/8 cells expressing the three FGFR3 mutants (Figure 2A). We also observed that the areas of Venus signals, which should be proportional to the number of Venus-positive cells, were increased by meclozine as well as by CNP in K650E- and G380R-expressing HCS-2/8 cells (Figure 2B).

### Meclozine mitigates abnormally suppressed differentiation of ATDC5 chondrogenic cells expressing FGFR3-G380R and -K650E in micromass culture

**Figure 3:**
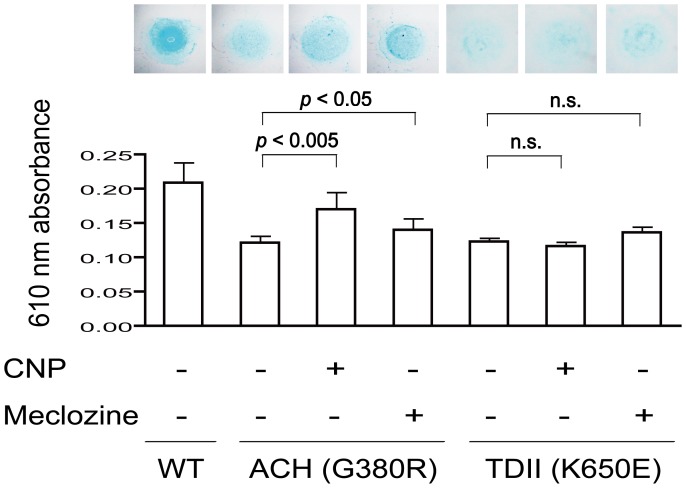
Meclozine facilitates chondrocyte differentiation of ATDC5 cells expressing mutant FGFR3 in micromass culture. ATDC5 cells were infected with lentivirus expressing FGFR3-WT (wild-type), FGFR3-G380R causing ACH, or FGFR3-K650E causing TDII. Similar expressions of the transgenes are shown by immunoblotting in Figure S3. ATDC5 cells in micromass culture were treated with CNP (0.2 µM) or meclozine (20 µM) for six days and were stained with Alcian blue. Experiments were repeated six times and representative images are shown. After staining, matrix proteoglycans in the cell lysate were quantified by measuring absorbance at 610 nm. The data are presented as the mean and SD (*n* = 6). CNP and meclozine increased the Alcian blue staining of ATDC5 cells expressing FGFR3-G380R and –K650E in micromass culture. Statistical significance is estimated by Student's t-test.

We next examined the effects of meclozine on chondrocyte differentiation in the presence of FGFR3 mutants. ATDC5 cells retain potency to differentiate into chondrocytes and are commonly used to study chondrogenesis *in vitro* [15]. We added meclozine to the micromass culture of ATDC5 cells that were infected with lentivirus expressing FGFR3-wild-type, FGFR3-G380R, or -K650E. Alcian blue staining revealed abundant sulfated proteoglycans on wild-type FGFR3 cells, while the staining intensity was reduced by the mutations. Addition of meclozine simultaneously with the chondrogenic induction alleviated the inhibitory effect of the G380R and K650E with and without statistical significance, respectively. Quantitative analysis of sulfated glycosaminoglycans in cell lysate demonstrated that meclozine increased the levels of glycosaminoglycans (Figure 3).

### Meclozine increases the longitudinal length of embryonic tibiae with or without FGF2 treatment in bone explant culture

**Figure 4:**
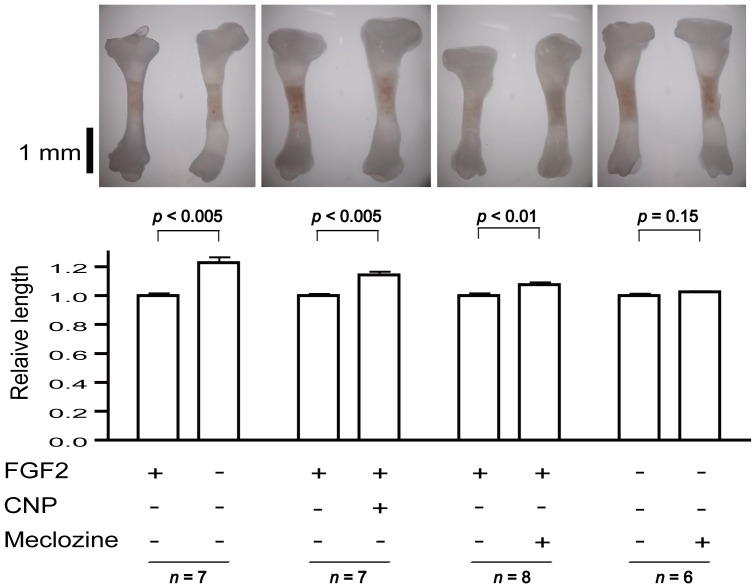
Meclozine increases the longitudinal length of embryonic tibiae with or without FGF2 treatment in bone explant culture. Unstained bilateral tibiae of the same individual were photographed side by side on day six of explant culture. Longitudinal bone lengths were normalized to that of untreated contralateral tibia, and the mean and SD are indicated. FGF2 causes inhibition of longitudinal bone growth. In the presence of FGF2, CNP (0.2 µM) and meclozine (20 µM) significantly increased the longitudinal length of embryonic tibiae. Even without FGF2, meclozine (20 µM) facilitated the growth of embryonic tibiae but without statistical significance (Student's t-test).

We further quantified the effect of meclozine on FGF2-mediated inhibition of cartilage development in the bone organ culture employing limb rudiments isolated from developing murine embryonic tibia [14]. We added combinations of 100 ng/ml FGF2, 0.2 µM CNP, and 20 µM meclozine to the culture medium, and compared the length of treated tibiae with that of contralateral control tibia from the same individual. The addition of FGF2 inhibited longitudinal growth of bone and cartilage of embryonic tibiae, while CNP and meclozine significantly attenuated the growth inhibition driven by FGF2 (Figure 4). Histological analysis revealed that FGF2 treatment reduced the thickness of the hypertrophic chondrocyte layer, while treatments with CNP and meclozine mitigated the effect of FGF2 (Figure S4). It is interesting to note that meclozine also increased the length of tibia without FGF2 treatment but without statistical significance.

### Meclozine attenuates ERK phosphorylation in FGF2-treated RCS cells

**Figure 5:**
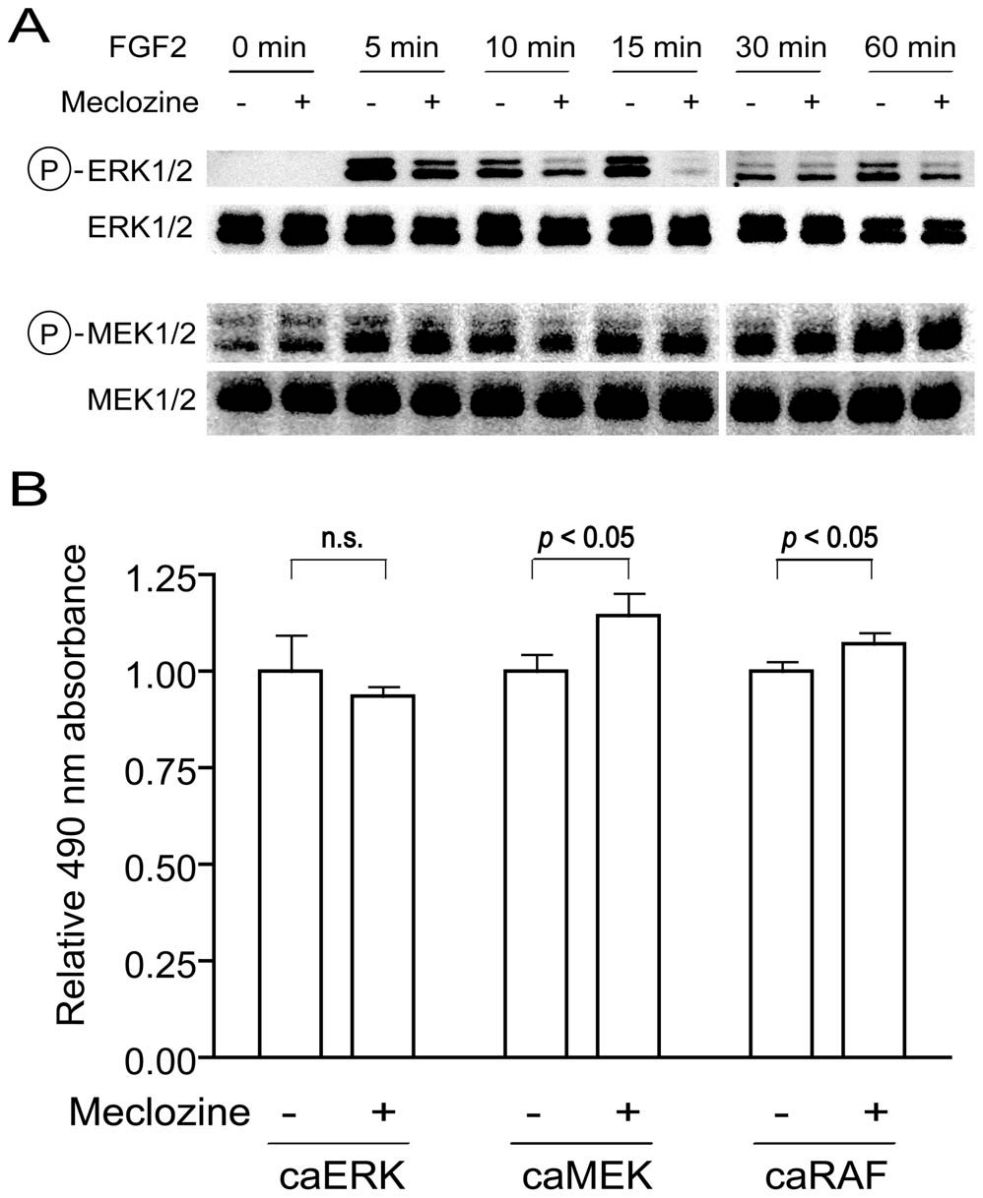
Meclozine attenuates FGFR3-mediated ERK phosphorylation in FGF2-treated RCS cells. (**A**) RCS cells were pretreated with 20 µM meclozine for 30 minutes before adding 5 ng/ml FGF2 and the levels of ERK and MEK phosphorylation were determined by Western blotting. As a loading control, the membranes were reprobed with antibodies against MEK and ERK. Meclozine suppressed FGF2-mediated ERK phosphorylation but not MEK phosphorylation after adding FGF2. (**B**) RCS cells were infected by lentivirus expressing constitutively active (ca) ERK, MEK, and RAF mutants. Cells were treated with 20 µM meclozine and their proliferation potencies were quantified using the MTS assay. The 490-nm absorbance was normalized to that without meclozine and the mean and SD are presented (*n* = 3). Meclozine rescued caMEK- and caRAF-mediated growth arrest, but had no effect on caERK-mediated growth arrest.

**Figure 6:**
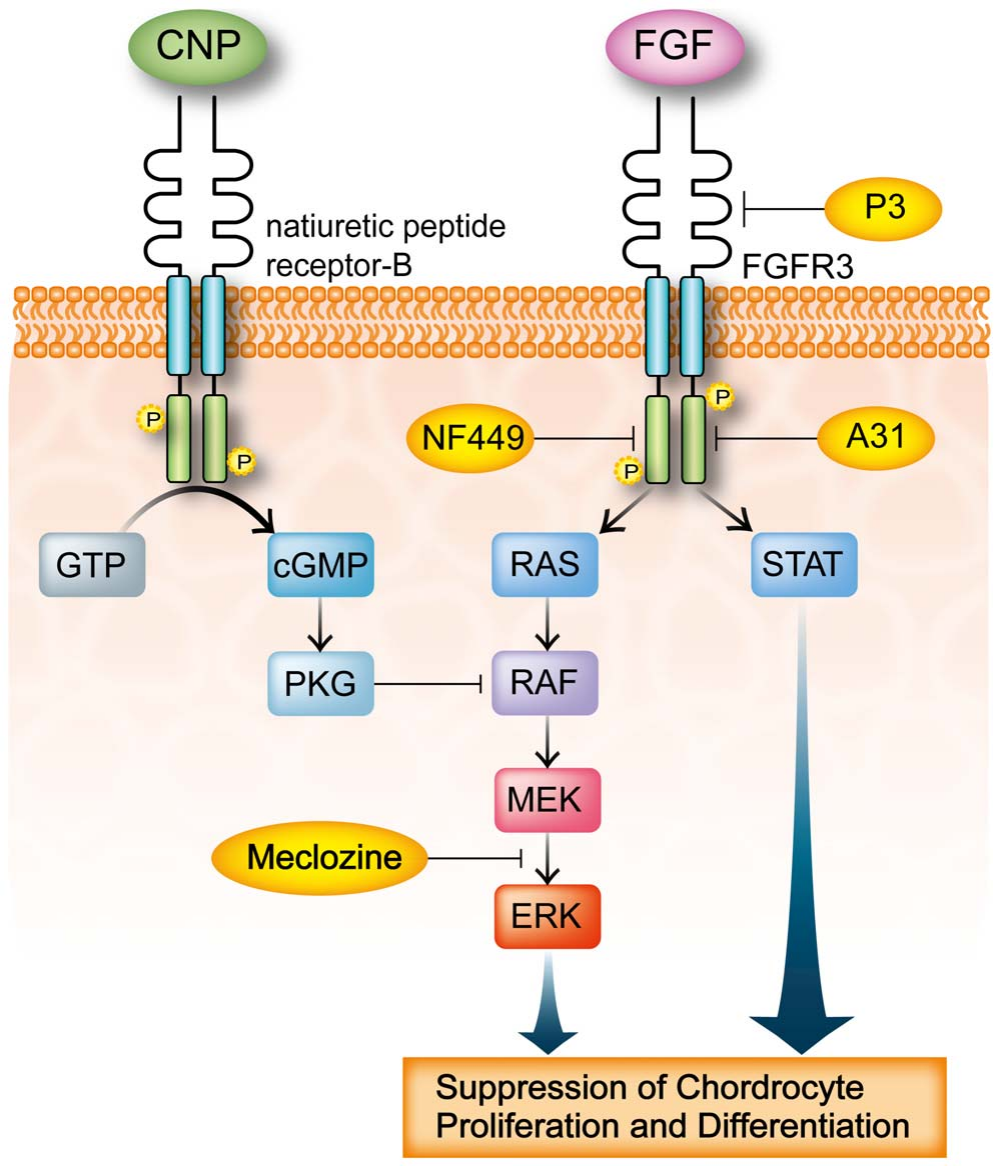
FGFR3 signal transduction in chondrocytes and mechanisms of FGFR3 inhibitors. Activations of MAPK (mitogen-activated protein kinase) and STAT (signal transducers and activators of transcription) negatively regulate chondrocyte proliferation and differentiation. MAPK signaling includes sequential stimulation of a signaling cascade involving RAS, RAF, MEK, and ERK. CNP binding to natriuretic peptide receptor-B induces the generation of the second messenger cGMP, which activates PKG and leads to attenuation of the MAPK pathway by inhibiting RAF activation. NF449 [14], A31 [16], and P3 [15] are recently identified FGFR3 inhibitors. NF449 and A31 have inhibitory effects on the kinase activity of FGFR3. P3 has an affinity for extracellular domain of FGFR3. Meclozine attenuates ERK phosphorylation.

We next scrutinized the effects of meclozine on the downstream signaling pathways of FGFR3 in FGF2-treated RCS cells. RCS cells were pretreated with meclozine for 30 minutes before adding FGF2, and the phosphorylation levels of ERK and MEK were determined by Western blotting. The FGF2-mediated ERK1/2 phosphorylation was attenuated by meclozine, while MEK1/2 phosphorylation remained unchanged (Figure 5A). We next introduced constitutively active (ca) mutants of ERK, MEK, and RAF into RCS cells using lentivirus and quantified cell growth with the MTS assay. As predicted, meclozine ameliorated caMEK- and caRAF-mediated growth inhibition, whereas meclozine had no effect on caERK-mediated growth inhibition (Figure 5B). We observed the similar effects by counting cells (Figures S5). Both data point to a notion that meclozine is likely to inhibit MEK1/2-mediated ERK1/2 phosphorylation or activate phosphatase(s) for phosphorylated ERK1/2 (Figure 6).

## Discussion

The drug repositioning strategy is an effort to identify new indications for the existing drugs. This strategy can potentially reduce the expenses and efforts associated with multi-stage testing of the hit compounds [20], [23]. Among the 1,186 FDA-approved drugs that have favorable or validated pharmacokinetic and toxicological profiles, we identified meclozine as a novel inhibitor of the FGFR3 signaling, which can potentially be applied to clinical practice for short stature in FGFR3-related skeletal dysplasias. Meclozine is an over-the-counter H1 blocker, which has been safely used for motion sickness for more than 50 years. Because the optimal doses and adverse effects of meclozine have already been established, meclozine can be readily prescribed for FGFR3-related skeletal dysplasia after effectiveness in humans is confirmed.

Since there is no rational therapy for FGFR3-related disorders available to date, development of novel modalities to suppress the FGFR3 signaling has long been expected. Krejci et al. screened a library of 1,120 compounds and identified that NF449 inhibits FGFR3 signaling in RCS chondrocytes as well as in FGF2-treated embryonic bone organ culture. NF449 is structurally similar to suramin and possesses inhibitory activities of other tyrosine kinases in addition to FGFR3 [14]. Jonquoyet et al. identified that a synthetic compound A31 is an inhibitor of the FGFR3 tyrosine kinase by *in silico* analysis. They demonstrated that A31 suppresses constitutive phosphorylation of FGFR3 and restores the size of embryonic femurs of *Fgfr3* ^Y367C/+^ mice in organ culture. In addition, A31 potentiates chondrocyte differentiation in the *Fgfr3* ^Y367C/+^ growth plate [16]. Jin et al. screened a library of random 12-peptide phages and found that P3 has a high and specific binding affinity for the extracellular domain of FGFR3. They showed that P3 promotes proliferation and chondrogenic differentiation of cultured ATDC5 cells, alleviates the bone growth retardation in bone rudiments from TD mice (*Fgfr3* ^Neo-K644E/+^ mice), and finally reversed the neonatal lethality of TD mice [15]. These novel FGFR3 tyrosine kinase inhibitors, however, may inhibit tyrosine kinases other than FGFR3 and may exert unexpected toxic effects in humans. Meclozine may also inhibit unpredicted tyrosine kinase pathways, but we can predict that there will be no overt adverse effect, because meclozine has been safely used for more than 50 years.

CNP is another therapeutic agent for FGFR3-related disorders. CNP-deficient mice were dwarfed with narrowing of the proliferative and hypertrophic zones of the growth plates [24]. Loss-of-function mutations in *NPR2* encoding a receptor for CNP are responsible for acromesomelic dysplasia Maroteaux-type (AMDM), a form of short-limbed human skeletal dysplasias [25]. Conversely, overexpression of CNP prevented the shortening of achondroplastic bones by inhibiting the MAPK signaling pathway [17]. Yasoda et al. demonstrated that continuous delivery of CNP through intravenous infusion successfully normalized the dwarfism of *Fgfr3* ^ach^ mice [18]. As CNP has a very short half-life, Lorget et al. developed an extended plasma half-life CNP analog, BMN111, which is resistant to neutral-endopeptidase digestion [19]. They showed that subcutaneous administration of BMN111 exhibits a significant recovery of bone growth in *Fgfr3* ^Y367C/+^ mice. Meclozine showed a similar inhibitory activity on the FGFR3 signaling compared to CNP in *ex vivo* bone explant culture as well as *in vitro* chondrogenic cells. We expect that meclozine can be used as a substitute for or in addition to CNP and the CNP analog.

The MAPK pathway is one of the major signaling pathways of FGFR3 in proliferation and differentiation of chondrocytes. Sustained ERK activation in chondrocytes leads to decreased proliferation, increased matrix degradation, altered cell shape, and decreased differentiation [5], [6]. CNP inhibits phosphorylation of RAF1 kinase through inhibition by PKGII [6], [17]. We demonstrated that meclozine attenuates ERK phosphorylation in chondrocytes. Gohil et al. reported that meclozine has an anti-oxidative phosphorylation (OXPHOS) activity in addition to anti-histamine and anti-muscarinic properties [26], [27]. In their report, meclozine showed cytoprotective activities against ischemic injury in the brain and heart. Since other drugs with anti-histamine, anti-muscarinic, and anti-OXPHOS properties did not show inhibition of the FGFR3 signaling in our studies, pharmacological actions of meclozine on chondrogenesis are unlikely to be relevant to its anti-histamine, anti-muscarinic, or anti-OXPHOS properties. Although additional studies are required to prove that meclozine is indeed effective for patients with FGFR3-related skeletal dysplasias, we propose that meclozine is an attractive and potential therapeutic agent.

## Materials and Methods

### Screening of 1,186 FDA-approved compounds in rat chondrosarcoma (RCS) cells

RCS cells, which were kindly provided from Dr. Pavel Krejci (Medical Genetics Institute, Cedars-Sinai Medical Center, LA) [5], were cultured in Dulbecco's Modified Eagle's Medium (DMEM, Invitrogen) supplemented with 10% fetal bovine serum (FBS, Thermo Scientific) [14]. For the RCS growth arrest assays, ∼5×10^3^ cells were seeded in a 96-well culture plate and incubated for 48 hours in the presence of 10 µM of 1,186 FDA-approved chemical compounds (Prestwick Chemical) and 5 ng/ml of FGF2 (R&D Systems). Cell proliferation was quantified by the MTS assay (Cell 96 AQueus One Solution Cell Proliferation Assay, Promega) according to the manufacturer's instructions. Cell numbers were counted using the TC 10 Automated Cell Counter (Bio-Rad). For counting cells, ∼1×10^5^ cells were seeded in a 12-well culture plate and incubated for 48 hours in the presence of 10 or 20 µM of meclozine and 5 ng/ml of FGF2.

### Alcian blue staining

For Alcian blue staining, ∼1×10^5^ RCS cells in a 12-well plate were added with 5 ng/ml FGF2 and also with either 10 µM meclozine or 0.2 µM CNP (Calbiochem). After 72 hours, cells were fixed with methanol for 30 minutes at −20°C, and stained overnight with 0.5% Alcian Blue 8 GX (Sigma) in 1 N HCl. For quantitative analyses, Alcian blue-stained cells were lysed in 200 µl of 6 M guanidine HCl for 6 hours at room temperature [28]. The optical density of the extracted dye was measured at 610 nm using PowerScan 4 (DS Pharma Biomedical).

### Total RNA extraction and real-time RT-PCR analysis

Total RNA was isolated from FGF2-treated RCS cells in the presence of 20 µM of meclozine or 0.2 µM of CNP using Trizol. The first strand cDNA was synthesized with ReverTra Ace (Toyobo). We quantified mRNA expression of matrix proteinases (*Mmp10*, *Mmp13*, and *Adamts1*) and extracellular matrix proteins (*Col2a1* and *Acan*) using LightCycler 480 Real-Time PCR (Roche) and SYBR Green (Takara). The mRNA levels were normalized for that of *Gapdh*.

### Vectors and cell transfection

The pRK7-FGFR3-WT, -K650E, and -K650M vectors expressing wild-type and mutant FGFR3 [29] were kindly provided by Dr. Pavel Krejci (Medical Genetics Institute, Cedars-Sinai Medical Center, LA). The pRK7-FGFR3-G380R was constructed by the QuikChange site-directed mutagenesis kit (Stratagene). The wild-type and mutant *FGFR3* cDNAs were excised from pRK7-FGFR3 vectors by double digestion with *Hin*dIII and *Bam*HI. The lentivirus vector, CSII-CMV-MCS-IRES2-Venus, was kindly provided by Dr. Hiroyuki Miyoshi (Riken BioResource Center, Tsukuba, Japan.), and was digested with *Nhe*I and *Bam*HI. The *Hin*dIII site of the insert and the *Nhe*I site of the vector were blunted using Quick Blunting Kit (New England Biolabs) before ligation. HEK293 cells were plated in a 150-mm dish on the day before transfection. We introduced the pLP1, pLP2, pLP/VSVG plasmids (ViraPower Packaging Mix, Invitrogen), and the CSII-CMV-MCS-IRES2-Venus vector into HEK293 cells with Lipofectamine 2000 (Invitrogen) according to the manufacturer's protocols. At 48 hours after transfection, we filtered the media containing the virus particles using the Millex-HV 0.45 µm PVDF filters (Millex) and purified lentivirus using two steps of ultracentrifugation (Beckman Coulter). The lentivirus was added to the medium of HCS-2/8 or ATDC5 cells. After 48 hours, we confirmed that more than 90% of cells were positive for Venus signals.

Clones that express constitutively active mutants in the MAPK/ERK pathway, pcDNA4Myc-ERK2(PD), pcDNA3HA-MEK1(DD), and pcDNA3Flag-C-rafΔN [30], were kindly provided by Dr. Mutsuhiro Takekawa (Medical Science Institute, Tokyo University, Japan). The inserts were digested with *Bam*HI and *Xho*I, and were cloned into the CSII-CMV-MCS-IRES2-Venus at the *Bam*HI sites after blunting all digested ends. Lentivirus particles were generated as described above and were used to infect RCS cells.

### Growth assay of human chondrosarcoma (HCS-2/8) cells

HCS-2/8 chondrocytic cells were kindly provided by Dr. Masaharu Takigawa [31]. The ethical review committee of Nagoya University Graduate School of Medicine approved the use of HCS-2/8 cells in condition that we do not analyze the whole genome of HCS-2/8 cells. HCS-2/8 cells were seeded in a 96-well tissue culture plate. HCS-2/8 cells were then infected with lentivirus expressing either FGFR3-WT, -K650E, -K650M, or G380R. After 48 hours, the numbers of cells were estimated by the MTS assay. In addition, ∼1×10^5^ HCS-2/8 cells in a 12-well tissue plate were introduced with lentivirus expressing FGFR3-G380R, or -K650E. After 72 hours, the Venus-positive cell areas were quantified by the ArrayScan VTI HCS Reader (Thermo Scientific).

### Micromass culture of ATDC5 cells

Mouse embryonic carcinoma-derived ATDC5 cells [32] were infected with lentivirus expressing either FGFR3-WT, -G380R, or -K650E. The infected ATDC5 cells were subjected to micromass cultures as described previously [33]. Briefly, ATDC5 cells were suspended in DMEM/F-12 (1∶1) medium (Sigma) containing 5% FBS at a density of 1×10^7^ cells/ml and plated in 10-µl droplets to simulate the high-density chondrogenic condensations. After 1-hour incubation, the same medium supplemented with 1% insulin-transferrin-sodium selenite (ITS, Sigma) was added to the cells. Medium was changed every other day until harvesting cells on day 6.

### Bone explant culture

The animal study was approved by the Animal Care and Use Committee of the Nagoya University Graduate School of Medicine. For bone explant culture, tibiae were dissected under the microscope from wild-type ICR mouse embryos on E16.5 (Japan SLC), placed in a 48-well plate, and cultured in BGJb medium (Invitrogen) supplemented with 0.2% bovine serum albumin and 150 µg/ml ascorbic acid. The medium was changed everyday. Embryonic tibiae were further treated with 100 ng/ml FGF2 in the presence or absence of 20 µM meclozine or 0.2 µM CNP for 6 days, then photographed and fixed in 10% formaldehyde in phosphate-buffered saline, demineralized with 0.5 M EDTA, and embedded in paraffin. Sections were stained with hematoxylin-eosin and Alcian blue. Images were taken with the SZ61 microscope (Olympus) equipped with the XZ-1 digital camera (Olympus). The longitudinal length of bone, which was defined as the length between proximal and distal articular cartilage, was measured using ImageJ (NIH).

### Western blotting and signaling studies

RCS cells were treated with or without 20 µM meclozine for 30 min before adding 5 ng/ml FGF2. After 0 to 60 min, cells were lysed in the ice-cold RIPA Lysis Buffer (Santa Cruz) supplemented with proteinase inhibitors. Whole cell lysates were separated on SDS-PAGE and transferred to a nitrocellulose membrane. The phosphorylation levels of molecules in the MAPK pathway were determined by Western blotting using the following antibodies: ERK1/2 (L34F12), phospho-ERK1/2(Thr^202^/Tyr^204^) (D13.14.4E), MEK1/2 (L38C12), and phospho-MEK1/2(Ser^217/221^) (41G9) purchased from Cell Signaling.

The expressions of the introduced wild-type and mutant FGFR3 constructs in HCS-2/8 and ATDC5 cells were determined by Western blotting using antibodies for FGFR3 (sc123, Santa Cruz) and GFP (11814460001, Roche).

## Supporting Information

Figure S1 **Expression levels of** ***Col2a1 and Acan*** **mRNAs were unchanged in FGF2-treated RCS cells.** Cells were treated with FGF2 for 72 hours and mRNAs were quantified by real-time RT-PCR. Expression levels of *Col2a1* and *Acan* mRNAs are presented as the mean and SD normalized to that of FGF2-negative cells (*n* = 3). FGF2 minimally suppressed the *Acan* expression but without statistical significance (Student's t-test).
(TIF)

Figure S2 **K650E-expressing HCS-2/8 cells showed suppressed cellular proliferation.** (**A**) Proliferation of HCS-2/8 cells infected with lentivirus expressing either FGFR3-WT (wild-type) or FGFR3-K650E (TDII) was quantified using the MTS assay at 48 hours after seeding. The growth of HCS-2/8 cells expressing FGFR3-K650E was significantly less than that of FGFR3-WT (*p*<0.001). The mean and SD are plotted. (**B**) Immunoblotting of FGFR3 and Venus to showing efficient expressions of FGFR3-WT and FGFR3-K650E in HCS-2/8 cells. FGFR3 is transcribed by CMV and Venus is downstream of IRES2 on the same transcript. As a control, the membrane was reprobed with Venus by anti-GFP antibody.
(TIF)

Figure S3 **Immunoblotting of FGFR3 showing efficient expressions of FGFR3-WT, -G380R, and -K650E in ATDC5 cells.** ATDC5 cells were infected with lentivirus expressing FGFR3-WT (wild-type), -G380R (ACH), and -K650E (TDII). FGFR3 is transcribed by CMV and Venus is downstream of IRES2 on the same transcript. As a control, the membrane was reprobed with Venus by anti-GFP antibody.
(TIF)

Figure S4 **Meclozine increases the thickness of embryonic tibial growth plate in FGF2-treated bone explant culture.** Tibia sections were stained with hematoxylin-eosin and Alcian blue on day six of explant culture. Arrows indicate hypertrophic chondrocyte layers. FGF2 treatment reduced the thickness of the layer, while treatments with CNP and meclozine mitigated the effect of FGF2.
(TIF)

Figure S5 **Meclozine attenuates FGFR3-mediated ERK phosphorylation in FGF2-treated RCS cells.** RCS cells were infected by lentivirus expressing constitutively active (ca) ERK, MEK, and RAF mutants. Cells were treated with 20 µM meclozine and the cell numbers were counted. The cell numbers were normalized to that without meclozine and the mean and SD are presented (*n* = 6). Meclozine rescued caMEK-mediated, but not caERK-mediated, growth arrest, although no statistical significance was observed.
(TIF)
