# Supplementary figures and images for: Meclozine Facilitates Proliferation and Differentiation of Chondrocytes by Attenuating Abnormally Activated FGFR3 Signaling in Achondroplasia
Source: PLoS One. 2013 Dec 4;8(12):e81569. doi: 10.1371/journal.pone.0081569 (PMC3852501; doi:10.1371/journal.pone.0081569)

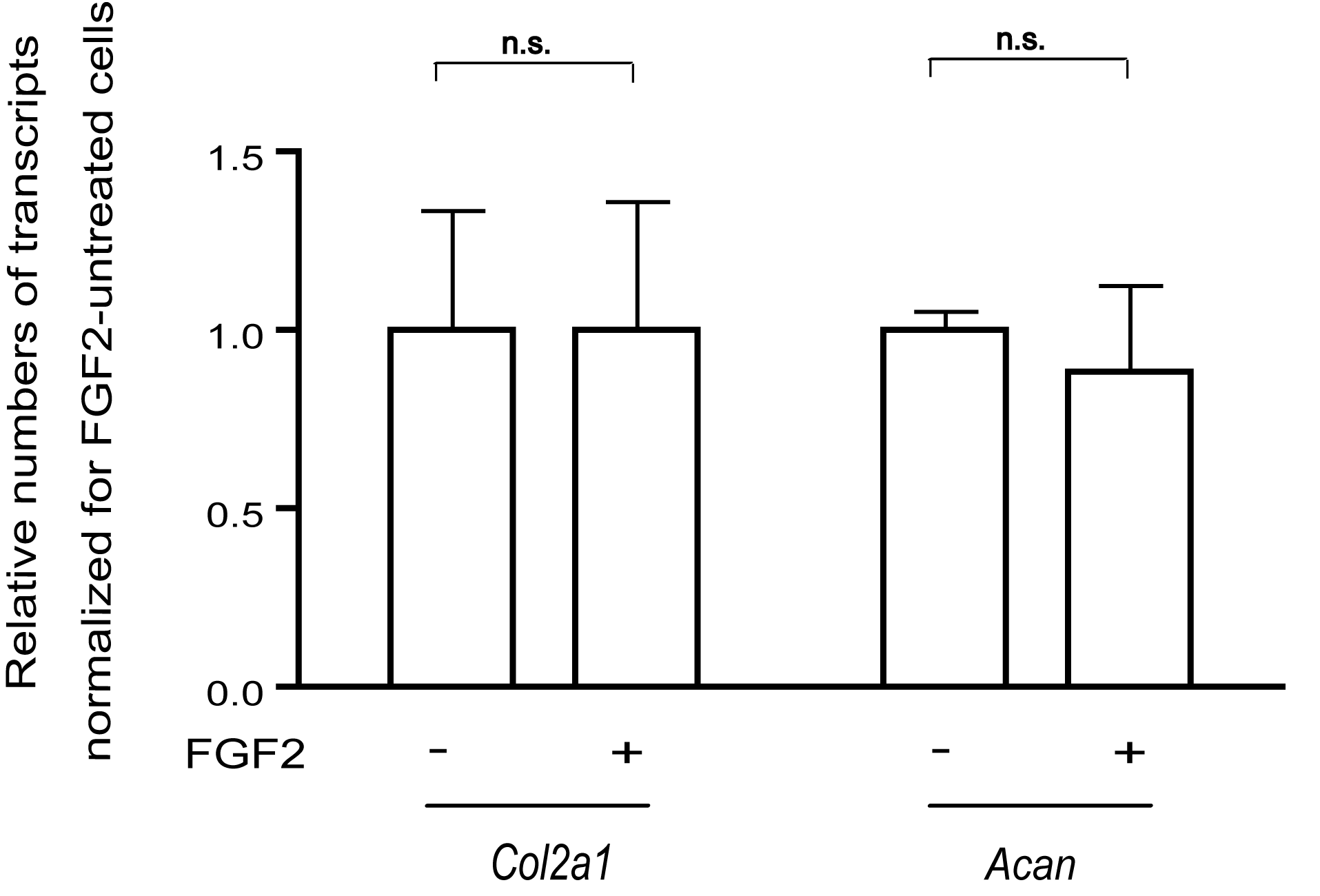

Supplement: Figure S1 — Expression levels of Col2a1 and Acan mRNAs were unchanged in FGF2-treated RCS cells. Cells were treated with FGF2 for 72 hours and mRNAs were quantified by real-time RT-PCR. Expression levels of Col2a1 and Acan mRNAs are presented as the mean and SD normalized to that of FGF2-negative cells (n = 3). FGF2 minimally suppressed the Acan expression but without statistical significance (Student's t-test). (TIF) [file pone.0081569.s001.tif]

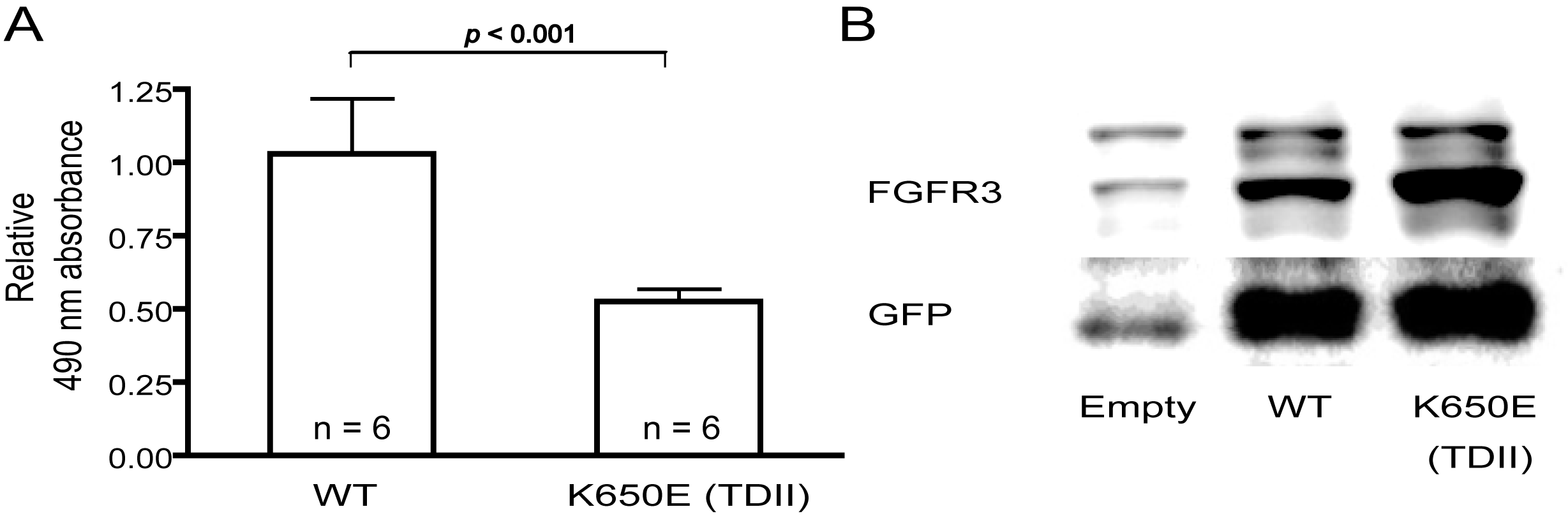

Supplement: Figure S2 — K650E-expressing HCS-2/8 cells showed suppressed cellular proliferation. (A) Proliferation of HCS-2/8 cells infected with lentivirus expressing either FGFR3-WT (wild-type) or FGFR3-K650E (TDII) was quantified using the MTS assay at 48 hours after seeding. The growth of HCS-2/8 cells expressing FGFR3-K650E was significantly less than that of FGFR3-WT (p<0.001). The mean and SD are plotted. (B) Immunoblotting of FGFR3 and Venus to showing efficient expressions of FGFR3-WT and FGFR3-K650E in HCS-2/8 cells. FGFR3 is transcribed by CMV and Venus is downstream of IRES2 on the same transcript. As a control, the membrane was reprobed with Venus by anti-GFP antibody. (TIF) [file pone.0081569.s002.tif]

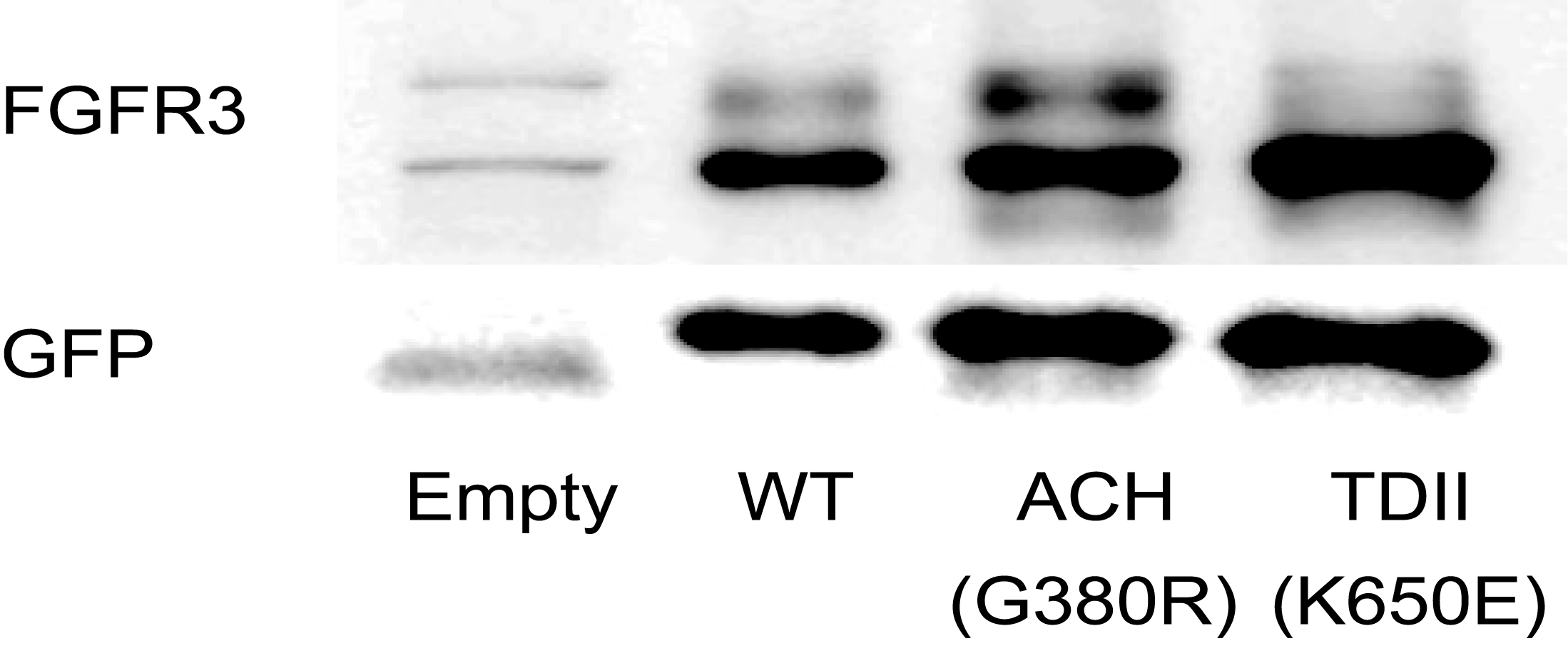

Supplement: Figure S3 — Immunoblotting of FGFR3 showing efficient expressions of FGFR3-WT, -G380R, and -K650E in ATDC5 cells. ATDC5 cells were infected with lentivirus expressing FGFR3-WT (wild-type), -G380R (ACH), and -K650E (TDII). FGFR3 is transcribed by CMV and Venus is downstream of IRES2 on the same transcript. As a control, the membrane was reprobed with Venus by anti-GFP antibody. (TIF) [file pone.0081569.s003.tif]

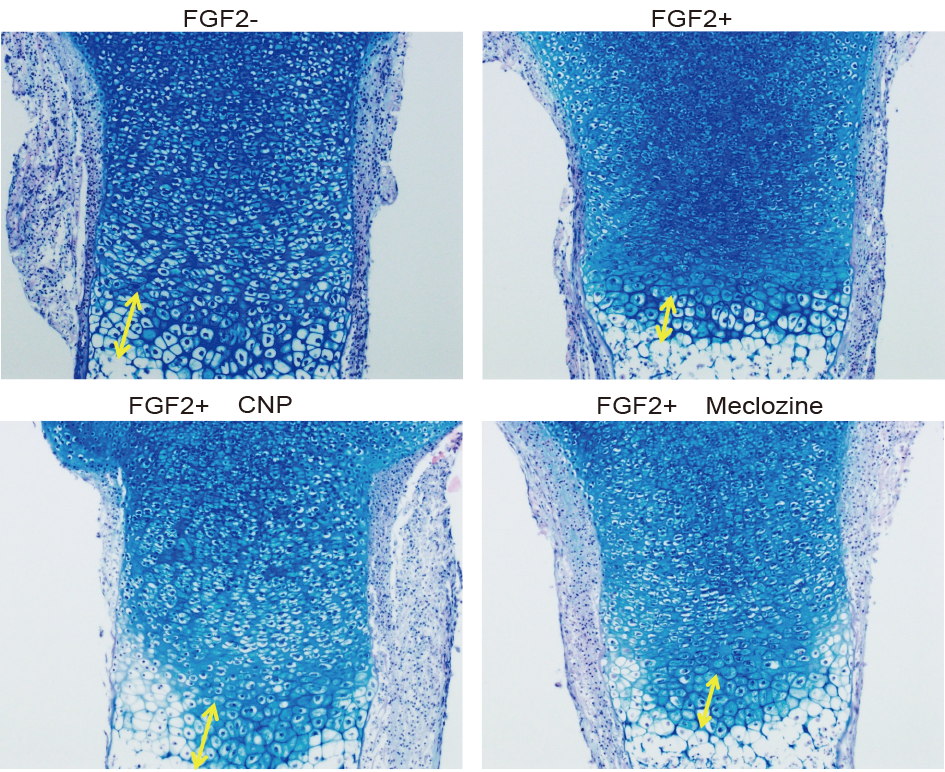

Supplement: Figure S4 — Meclozine increases the thickness of embryonic tibial growth plate in FGF2-treated bone explant culture. Tibia sections were stained with hematoxylin-eosin and Alcian blue on day six of explant culture. Arrows indicate hypertrophic chondrocyte layers. FGF2 treatment reduced the thickness of the layer, while treatments with CNP and meclozine mitigated the effect of FGF2. (TIF) [file pone.0081569.s004.tif]

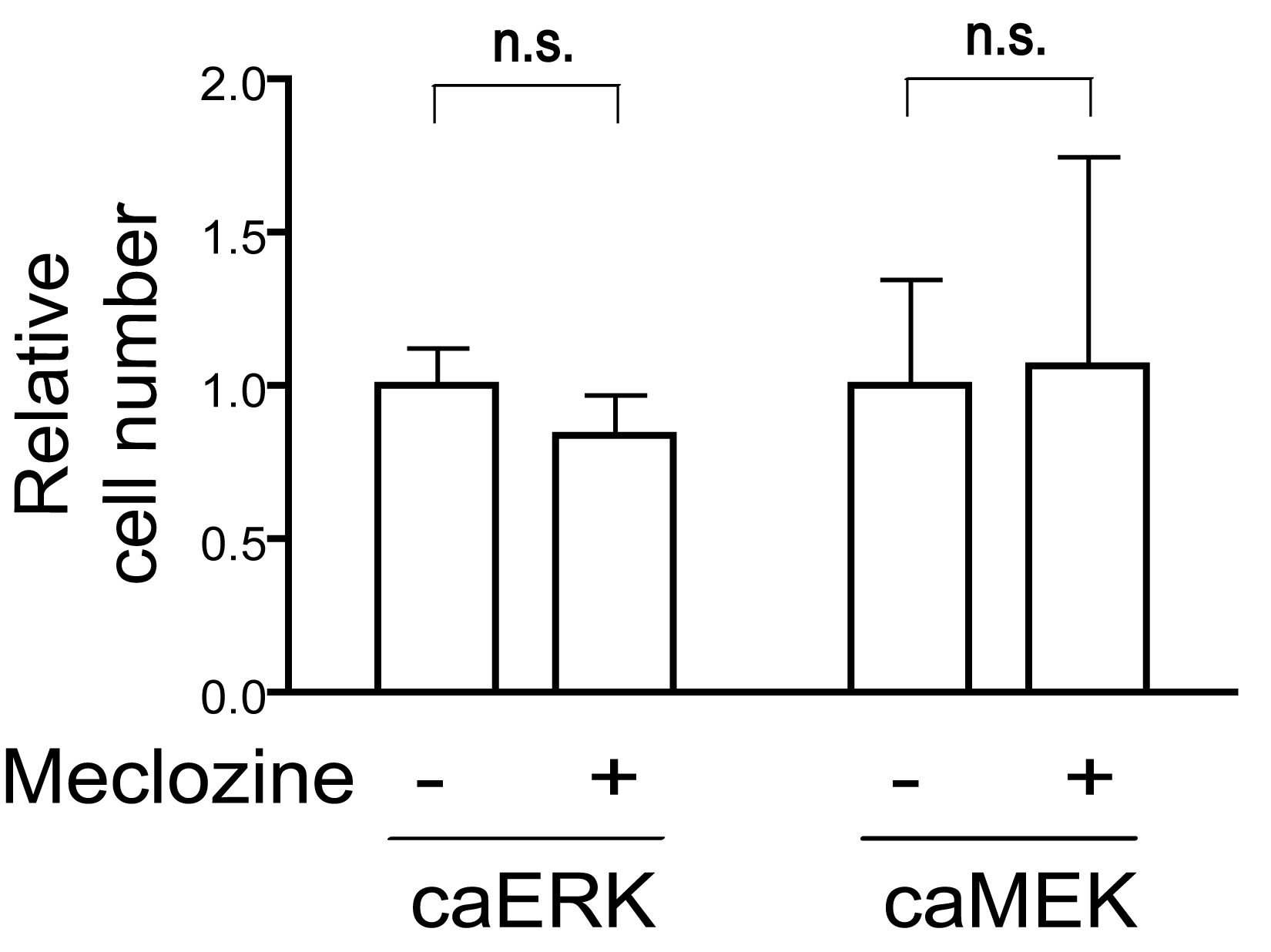

Supplement: Figure S5 — Meclozine attenuates FGFR3-mediated ERK phosphorylation in FGF2-treated RCS cells. RCS cells were infected by lentivirus expressing constitutively active (ca) ERK, MEK, and RAF mutants. Cells were treated with 20 µM meclozine and the cell numbers were counted. The cell numbers were normalized to that without meclozine and the mean and SD are presented (n = 6). Meclozine rescued caMEK-mediated, but not caERK-mediated, growth arrest, although no statistical significance was observed. (TIF) [file pone.0081569.s005.tif]
